# Oral prednisolone suppresses skin inflammation in a healthy volunteer imiquimod challenge model

**DOI:** 10.3389/fimmu.2023.1197650

**Published:** 2023-07-20

**Authors:** Salma Assil, Thomas P. Buters, Pieter W. Hameeteman, Charlie Hallard, Nicoline Treijtel, Tessa Niemeyer – Van der Kolk, Marieke L. de Kam, Edwin F. I. I. I. Florencia, Errol P. Prens, Martijn B. A. van Doorn, Robert Rissmann, Naomi B. Klarenbeek, Manon A. A. Jansen, Matthijs Moerland

**Affiliations:** ^1^ Centre for Human Drug Research, Leiden, Netherlands; ^2^ Division of Biotherapeutics, Leiden Academic Centre for Drug Research, Leiden University, Leiden, Netherlands; ^3^ Leiden University Medical Centre, Leiden, Netherlands; ^4^ Department of Dermatology Erasmus Medical Centre, Rotterdam, Netherlands

**Keywords:** TLR7, imiquimod, corticosteroids, inflammatory model, healthy volunteer

## Abstract

Imiquimod (IMQ) is a topical agent that induces local inflammation *via* the Toll-like receptor 7 pathway. Recently, an IMQ-driven skin inflammation model was developed in healthy volunteers for proof-of-pharmacology trials. The aim of this study was to profile the cellular, biochemical, and clinical effects of the marketed anti-inflammatory compound prednisolone in an IMQ model. This randomized, double-blind, placebo-controlled study was conducted in 24 healthy volunteers. Oral prednisolone (0.25 mg/kg/dose) or placebo (1:1) was administered twice daily for 6 consecutive days. Two days after treatment initiation with prednisolone or placebo, 5 mg imiquimod (IMQ) once daily for two following days was applied under occlusion on the tape-stripped skin of the back for 48 h in healthy volunteers. Non-invasive (imaging and biophysical) and invasive (skin punch biopsies and blister induction) assessments were performed, as well as IMQ *ex vivo* stimulation of whole blood. Prednisolone reduced blood perfusion and skin erythema following 48 h of IMQ application (95% CI [−26.4%, −4.3%], p = 0.0111 and 95% CI [−7.96, −2.13], p = 0.0016). Oral prednisolone suppressed the IMQ-elevated total cell count (95% CI [−79.7%, −16.3%], p = 0.0165), NK and dendritic cells (95% CI [−68.7%, −5.2%], p = 0.0333, 95% CI [−76.9%, −13.9%], p = 0.0184), and classical monocytes (95% CI [−76.7%, −26.6%], p = 0.0043) in blister fluid. Notably, TNF, IL-6, IL-8, and Mx-A responses in blister exudate were also reduced by prednisolone compared to placebo. Oral prednisolone suppresses IMQ-induced skin inflammation, which underlines the value of this cutaneous challenge model in clinical pharmacology studies of novel anti-inflammatory compounds. In these studies, prednisolone can be used as a benchmark.

## Introduction

Early phase clinical research commonly evaluates the safety, tolerability, and pharmacological activity of novel compounds (1–3). For anti-inflammatory and immunomodulatory compounds, immune challenge models are becoming increasingly popular to demonstrate ‘proof of pharmacology’ at an early clinical stage, thereby providing insight into the mechanism of action and providing target engagement (4). These pharmacological challenge models are often translated from animal work and can guide drug developers on dose selection and dosing regimens for subsequent phase II studies in the target population.

A widely used preclinical model to study (modulation of) inflammation, also applied in healthy volunteers (HV), is the topical imiquimod (IMQ) challenge, which drives a toll-like receptor (TLR) 7-mediated response. The TLR-dependent pathway activates nuclear factor kappa B (NF-κB) signaling and IRF *via* My-D88, which is important for an early immune response, such as secretion of pro-inflammatory cytokines, including interferon (IFN) -α, interleukin (IL)-1, IL-1RA, IL-6, and IL-8 (5). IMQ applied under occlusion for 48 h drives a transient local reversible inflammatory response indicated by an increase in blood perfusion, erythema, and cytokine production (6). The IMQ model is valuable for the evaluation of the potential combined effect of IMQ and omiganan (a synthetic indolicidin) in HV (7). The IMQ challenge can be valuable for proof-of-mechanism studies of compounds that target TLR7-mediated responses. However, formal benchmarking of the topical IMQ challenge model in humans, using a registered anti-inflammatory drug has not yet been performed.

An alternative human innate immune challenge model is intradermal LPS challenge, which drives TLR4-mediated responses. Corticosteroid treatment (oral prednisolone and topical clobetasol propionate) suppressed the characteristics of the dermal inflammatory reaction, which was also reflected by a reduction in inflammatory cell attraction in the blister fluid (8, 9). Moreover, for the topical IMQ model, profiling of effects of a known strong anti-inflammatory compound that is widely used in dermatology, such as oral prednisolone, would be valuable to benchmark the model for future evaluations of novel anti-inflammatory or immunomodulatory compounds.

Therefore, in this study, we aimed to profile the cellular, biochemical and clinical effects of oral prednisolone on the IMQ skin inflammation model in healthy volunteers with the goal of i) benchmarking the IMQ model for future novel anti-inflammatory compounds, ii) expanding the mechanistic insights into the IMQ-driven skin response by a more thorough molecular and cellular evaluation, and iii) gaining insight into the immunomodulatory mechanism of prednisolone in TLR7-mediated tissue inflammation.

## Materials and methods

This randomized, double-blind, placebo-controlled, investigator-initiated study was conducted according to the Dutch Act on Medical Research involving Human Subjects (WMO), and the study protocol was approved by the Medical Ethics Committee (Stichting Beoordeling Ethiek Biomedisch Onderzoek, Assen, The Netherlands) prior to the start of the clinical phase. The subjects provided written informed consent before any study-related procedures were undertaken.

### Study design and subjects

A total of 24 healthy male and female Caucasian (Fitzpatrick skin type I–III) volunteers, aged between 18 and 45 years, were enrolled in this study. Health status was confirmed by medical history, physical examination, laboratory tests, and 12-lead electrocardiography (ECG). None of the participants had a family history of psoriasis, no pathological skin conditions at the treatment area, no history of hypertrophic scarring or keloids, no prior use of imiquimod, and no known hypersensitivity to prednisolone.

### Treatment and IMQ challenge

**Figure 1:**
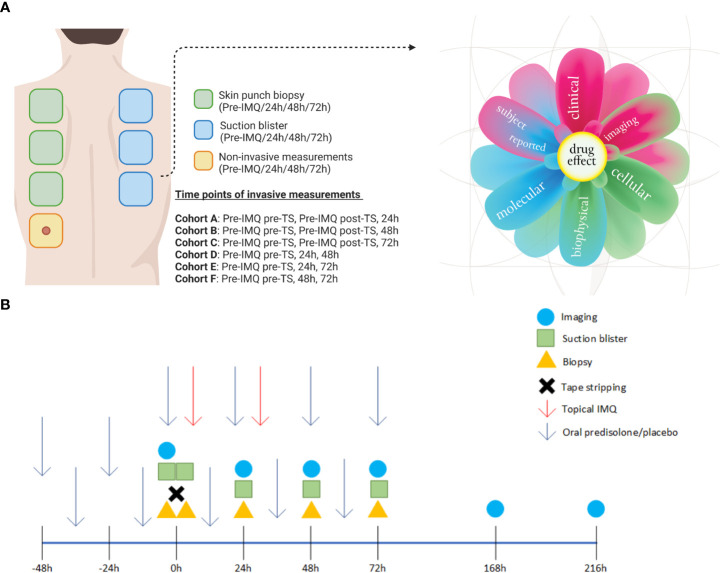
Overview of the treated sites on the back and study schedule. **(A)** A total of three biopsies and three blisters were obtained from each subject, with the timing and location of each procedure determined by their assigned cohort. One site on the back was used solely for non-invasive measurements throughout the study period. The skin responses were evaluated using a multi-modal approach and are represented in the “derma flower.” **(B)** A schematic overview of all assessments performed relative to dosing. Created with BioRender.com. Derma flower created by F. van Meurs, adapted for this manuscript.

Participants were equally randomized into two groups to receive oral prednisolone (0.25 mg/kg/dose) or placebo, twice daily with a 10–12-hour interval between doses, over a period of six consecutive days. On the sixth day, the volunteers received only one dose of prednisolone or placebo in the morning. The treatments were administered under supervision at the clinical research unit to ensure treatment compliance. After two days of pre-treatment with oral prednisolone or placebo, challenge with IMQ commenced (6). For this purpose, the upper back was divided into seven rectangles measuring 4 × 3 cm. Each treatment area was tape-stripped 20 times (D-Squame, CuDerm, Dallas, TX) to induce mild barrier skin disruption, whereas the trans-epidermal water loss (TEWL) (AquaFlux, Biox Systems) was used to quantify skin permeability (6, 7). A TEWL between 15 and 20 g/m^2^/h was considered as mild barrier skin disruption. No IMQ was applied to the first two areas since they represented the non-treated areas, while in the other three to five areas, 5 mg IMQ (100 mg Aldara^®^) was applied for either 24 h or 48 h (depending on the assigned cohort) under occlusion by a 12 mm Finn chamber (Bipharma, Almere, The Netherlands) to initiate an inflammatory skin reaction. Subjects were randomized on (pre-)treatment (prednisolone or placebo) and the time point of invasive measurements (blister and biopsy), as illustrated in **Figure 1**.

### Imaging-based endpoints

The subjects underwent multiple assessments to evaluate the inflammatory skin response before the IMQ challenge and 24, 48, 72, 168, and 216 h after IMQ application. A single treatment site was selected to evaluate non-invasive endpoints throughout the study period (**Figure 1**), resulting in N = 12 per time point. An overview of the number of samples per time-point is presented in **Table S1**. Erythema was graded in two ways: by a physician using a 4-point scale ranging from 0 (absent) to 3 (severe) and by multispectral photoanalysis (Antera 3D, Miravex, Ireland). Perfusion was quantified using laser speckle contrast imaging (LSCI; PeriCam PSI System, Perimed Jäfälla, Sweden) and Optical Coherence Tomography (OCT; VivoSight, Michelson Diagnostics Maidstone, UK). The latter was also used to measure epidermal thickness. All skin assessments were performed under standardized conditions at room temperature (20–24°C).

### Biopsy and blister exudate assessments

Suction blisters and 3 mm biopsy samples were taken from the IMQ-treated areas and control areas at the indicated time points, depending on the cohort and randomization (**Figure 1**). Three biopsies and three blisters were collected from each healthy volunteer. Suction blisters were induced according to the method described by Buters et al. (8). Biopsies were placed in RNAlater medium directly after harvest and stored at 4°C until analysis at the Immunology Laboratory of Erasmus Medical Center, Rotterdam, Netherlands. Immunohistochemical staining was performed to obtain scoring of markers CD11c (Clone 5D11; Cell Marque), CD14 (Clone EPR3653; Cell Marque), CD1a (Clone EP3622; Cell Marque), CD4 (Clone SP35; Ventana), CD8 (Clone SP57; Ventana), and HLA-DR (CR3/43; Dako) using a 6-point rating scale; 0 = negative, 1 = minimal, 2 = few, 3 = moderate, 4 = many, and 5 =excessive.

Blister fluid was collected in a V-bottom plate containing 50 μl 3% sodium citrate (Sigma) in PBS (Gibco) and kept on ice. The plate was centrifuged, and the supernatant was weighed to estimate the volume and then frozen at −80°C for cytokine analysis (Meso Scale Discovery, Rockville, Maryland, USA). The following cytokines were analyzed: TNF, ASC, IL-1β, IL-6, IL-10, IL-8, IFN-γ, and downstream marker for type 1 interferon Mx-A (V-plex proinflammatory panel of MSD and Mx-A protein ELISA kit from BioVendor). The cell pellet was resuspended in RoboSep buffer (StemCell). A cocktail of fluorescent antibodies against cell surface markers was added to the cells and incubated for 30 min on ice. The stained samples were washed with PBS and measured using MACSQuant 16 (Miltenyi Biotec GmbH). Flow cytometry data were analyzed with Flowlogic 7.2 (Inivai). Parallel to the blister fluid, peripheral blood was collected by venipuncture using a sodium heparin vacutainer (BD). Approximately 100 µl of whole blood was treated with red blood cell lysis buffer (eBioscience), washed with PBS, and resuspended in RoboSep buffer. Staining was similar to that of the previously mentioned blister cells and served as a template for the gating strategy. The following antibodies were used: CD56-PE (cat# 130-113-312, Miltenyi Biotec), CD14-PE-Vio615 (cat# 130-110-526, Miltenyi Biotec), CD16-VioBrighT FITC (cat# 130-119-616, Miltenyi Biotec), CD66b-AF700 (cat# 305114, Biolegend), CD19-BV650 (cat# 302238, Biolegend), CD20-BV650 (cat# 302336, Biolegend), HLA DR-APC (cat# 130-111-790, Miltenyi Biotec), CD4-VioBlue (cat# 130-114-534, Miltenyi Biotec), CD8-BV570 (cat# 301038, Biolegend), CD45-VioGreen (cat# 130-110-638, Miltenyi Biotec), CD1c-PE-Vio770 (cat# 130-110-538, Miltenyi Biotec), CD3-APC-Vio770 (cat# 130-113-136, Miltenyi Biotec), 7AAD (cat# 130-111-568, Miltenyi Biotec). An overview of the gating strategy is shown in **Figure S2**. Cell populations (Single live cells) were classified according to the following profile: CD45^+^ HLA-DR^−^ CD66b^+^ CD16^+^ neutrophils, CD45^+^ HLA-DR^+^ CD14^+^ CD16^−^ classical monocytes, CD45^+^ HLA-DR^+^ CD14^+^ CD16^+^ intermediate monocytes, CD45^+^ HLA-DR^+^ CD14^−^ CD16^+^ non-classical monocytes, CD45^+^ HLA-DR^+^ CD19^−^ CD20^−^ CD14^−^ CD16^−^ CD1c^+^ dendritic cells, CD45^+^ HLA-DR^−^ CD56^+^ NK Cells, CD45^+^ HLA-DR^−^ CD3^+^ CD4^+^ CD8^−^ T helper cells, CD45^+^ HLA-DR^−^ CD3^+^ CD4^−^ CD8^+^ cytotoxic T cells, and CD45^+^ HLA-DR^+^ CD19^+^ CD20^+^ B cells.

### *Ex vivo* whole blood stimulation

To investigate the extent of systemic immune suppression with prednisolone (*ex vivo* drug activity), IMQ whole-blood stimulation was used with cytokine release as a readout. Blood was drawn from healthy volunteers at four time points: pre-dose, 48, 52, and 96 h after the initial prednisolone/placebo dose. Blood samples were drawn 48 h and 96 h after initial dose but before the morning prednisolone/placebo dose. The sample taken 52 h after the first administration was taken 4 h after the previous prednisolone/placebo dose. At these time points, sodium heparinized whole blood was stimulated with 20 µg/ml IMQ (cat# tlrl-imq, InvivoGen) for 24 h. After incubation, the cultures were spun down, and the supernatant was collected and frozen at −80°C for cytokine analysis. The samples were shipped to Ardena (Assen, Netherlands) for analysis. The following cytokines were analyzed: IL-1β, IL-6, IFN-γ (V-plex proinflammatory panel of MSD), IP-10 (V-plex chemokine panel of MSD), and Mx-A (human Mx-A protein ELISA kit from BioVendor).

### Statistics

All repeatedly measured PD endpoints were summarized (n, mean, SD, min, and max values) according to treatment and time. Repeatedly measured continuous PD endpoints were analyzed using a mixed model analysis of covariance with fixed factor treatment, time, and treatment by time, with a random factor subject as a covariate. The baseline for whole blood stimulation was the pre-prednisolone/placebo treatment measurement. A summary table of the analysis results per variable was generated with estimates of the difference of the different contrasts and a back-transformed estimate of the difference in percentage for log-transformed parameters, 95% confidence intervals (in percentage for log-transformed parameters), Least Square Means (geometric means for log transformed parameters), and the p-value of the contrasts. Statistical analyses were performed using SAS for Windows V9.4 M6 (SAS Institute Inc., Cary, NC, USA).

## Results

Twenty-one female (87.5%) and three male (12.5%) Caucasian subjects participated in the study and completed the study without withdrawal. The mean age was 26.3 ± 4.6 years. Headache was the most frequent adverse event; however, it was mild in nature and probably related to the administration of prednisolone. No serious adverse events were reported during the study.

### Oral corticosteroids suppress the clinical response induced by topical IMQ application

**Figure 2:**
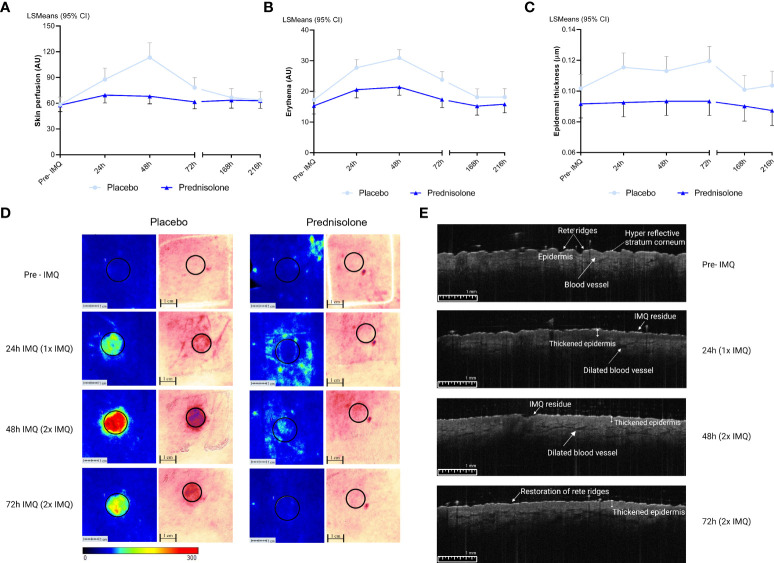
Imaging and biophysical based assessments. Pre-IMQ timepoint refers to assessment prior to IMQ application, whereas 24h refers to 1x IMQ application and 48h to 2x IMQ application. 72h refers to 2x IMQ application however measured 24 hours post last IMQ application. For the following measurements prednisolone is compared to placebo. **(A)** Skin perfusion by LSCI, estimated difference: -16.1%, 95% CI [-26.4%, -4.3%], p= 0.0111. **(B)** Erythema measured by multispectral camera, estimated difference: -5.04, 95% CI [-7.96, -2.13], p= 0.0016. **(C)** Epidermal thickness by OCT, estimated difference: -0.018, 95% CI [-0.029, -0.006], p= 0.0044. **(D)** A visual overview of LSCI and multispectral imaging in the prednisolone and placebo group. A ROI of 12 mm was selected for quantification. **(E)** OCT image of one subject of the placebo group after application of IMQ over time. CI, confidence interval; IMQ, Imiquimod; LSCI, Laser speckle contrast imaging; LSMeans, Least Squares Mean; OCT, Optical coherence tomography.

Oral prednisolone or placebo was administered at 0.25 mg/kg per dose b.i.d. for a period of six consecutive days, and the effects on IMQ-driven clinical responses were evaluated. IMQ was applied for a maximum of 48 h under occlusion, after the skin was tape-stripped 20 times. Maximal responses were observed 48 h after IMQ application in the placebo group (**Figures 2A, B**). Treatment with prednisolone resulted in a reduction in the IMQ-driven response from 24 h to 72 h, quantified by imaging of blood perfusion (estimated difference: −16.1%, 95% confidence interval (CI) [−26.4%, −4.3%], p = 0.0111), erythema (estimated difference: −5.04, 95% CI [−7.96, −2.13], p = 0.0016), and epidermal thickness (estimated difference: −0.018, 95% CI [−0.029, −0.006], p = 0.0044), compared to placebo (**Figures 2A-C**). A visual overview of the blood perfusion and erythema is shown in **Figure 2D**. Application of IMQ (for 24 and 48 h) on the skin resulted in a thickened epidermis, disappearance of the rete ridges and dilatation of the blood vessels (**Figure 2E**). Furthermore, prednisolone treatment shifted the clinically scored erythema response from moderate to mild (at 48 h) and reduced the proportion of clinically scored erythema (**Figure S1**). All quantified responses (perfusion, erythema, and epidermal thickness) were reversible and returned to baseline during the follow-up phase (168 and 216 h).

### Oral corticosteroids reduce the IMQ-driven cell infiltration in blister exudate and biopsy

In earlier skin challenge research, cellular responses were studied using invasive techniques including suction blisters and skin punch biopsies (6–9). In this study, we implemented the same approach by inducing suction blisters and taking skin punch biopsies at the indicated time points, resulting in a total of three blisters and three biopsies per subject (**Figure 1**). Biopsy samples were stained for dermal immune cell infiltration and scored by an independent investigator blinded to the treatment.

**Figure 3:**
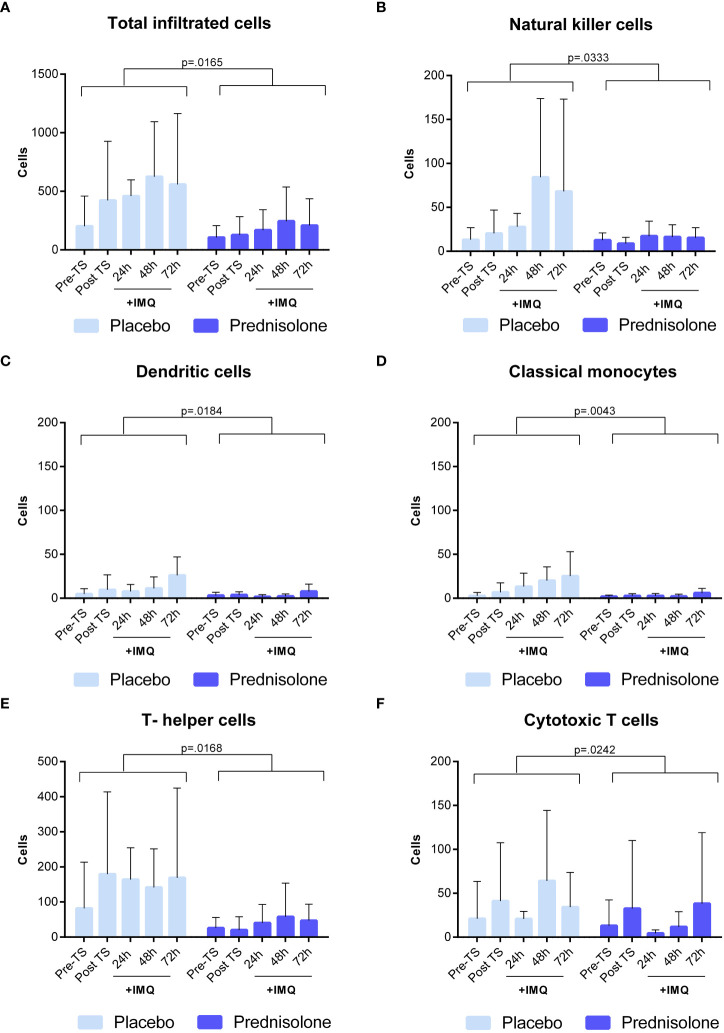
Oral prednisolone reduces IMQ- driven immune cell infiltration. **(A)** Total cells **(B)** Natural killer cells **(C)** Dendritic cells **(D)** Classical monocytes **(E)** T helper cells **(F)** Cytotoxic T cells. Immune cells in blister exudate were quantified by flow cytometry at different time points, pre and post IMQ application. Data are presented as mean ± SD. SD, standard deviation; TS, tape stripped.

In addition, immune cells in the blister exudate were evaluated using flow cytometry. A full overview of the analyzed immune cell subsets is shown in **Figure 3**. The average time for blister induction was (estimated mean, 95% CI) 86.7 min, 95% CI [71.9, 101.4] in the placebo group and 84.1 min, 95% CI [69.4, 98.8] in the prednisolone group (data not shown). No significant difference in the time required for blister formation was observed between the groups (estimated difference: −2.5, 95% CI [−23.4, 18.3], p = 0.8034). The total number of cells (CD45^+^) increased mildly following IMQ application, with a peak of 624.6 ± 469.5 cells in blister exudate at 48 h (after 2× IMQ application) (**Figure 3A**). IMQ increased the number of CD45^+^ HLA-DR^−^ CD56^+^ (NK cells), reaching a maximum of 84.2 ± 89.76 cells also at 48 h (after 2× IMQ application) (**Figure 3B**). The infiltration was followed by other innate immune cells, such as CD45^+^ HLA-DR^+^ CD19^−^ CD20^−^ CD14^−^ CD16^−^ CD1c^+^ (dendritic cells) and CD45^+^ HLA-DR^+^ CD14^+^ CD16^−^ (“classical monocytes”) peaking at 72 h (24 h after the second IMQ application) with mean ± SD of 26.0 ± 21.06 cells and 25.3 ± 27.7 cells, respectively; however, this resulted in a weaker response compared to the influx of NK cells (**Figures 3C, D**). Although neutrophils are strongly involved in innate immune responses, no infiltration of CD45^+^ HLA-DR^−^ CD66b^+^ CD16^+^ (neutrophils) was observed after IMQ application, resulting in cell counts of <5 cells (data not shown). Similar low cell counts were observed for CD45^+^ HLA-DR^+^ CD14^+^ CD16^+^ (“intermediate monocytes”), CD45^+^ HLA-DR^+^ CD14^−^ CD16^+^ (“non-classical monocytes”), and CD45^+^ HLA-DR^+^ CD19^+^ CD20^+^ (B cells) (data not shown). IMQ treatment did not result in a significant increase in B cells in the blister fluid (data not shown), nor did it substantially attract CD45^+^ HLA-DR^−^ CD3^+^ CD4^+^ CD8^−^ (T helper cells) and CD45^+^ HLA-DR^−^ CD3^+^ CD4^−^ CD8^+^ (cytotoxic T cells) (**Figures 3E, F**). Prednisolone reduced the number of immune cells in blister fluid. This reduction, compared to placebo, was observed for total cells, NK cells, dendritic cells, and classical monocytes (−58.8%, 95% CI [−79.7%, −16.3%], p = 0.0165; −45.5%, 95% CI [−68.7%, −5.2%], p = 0.0333; −55.4%, 95% CI [−76.9%, −13.9%], p = 0.0184; and −58.6%, 95% CI [−76.7%, −26.6%], p = 0.0043, respectively). Although no substantial changes were observed following IMQ challenge, prednisolone significantly reduced the number of T cell subsets (estimated difference: −76.0%, 95% CI [−92.4%, −4.7%], p = 0.0168 for T helper cells and estimated difference: −70.5%, 95% CI [−89.6%, −16.0%], p = 0.0242) for cytotoxic T cells.

**Figure 4:**
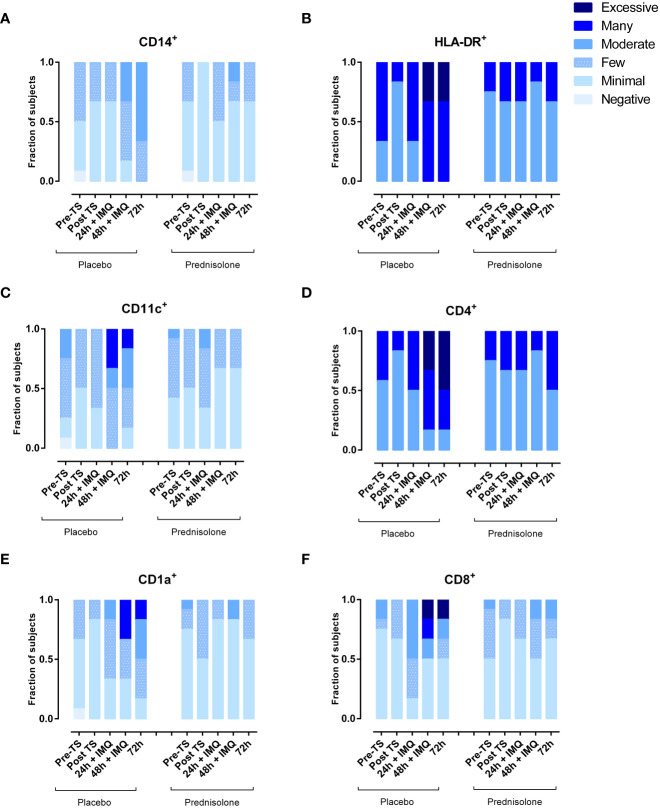
Prednisolone reduces infiltration of IMQ-driven immune cells as measured by IHC in skin punch biopsies. **(A)** Macrophages **(B)** HLA-DR **(C)** myeloid dendritic cells **(D)** T helper cells **(E)** Langerhans cells **(F)** Cytotoxic T cells. TS, tape stripped.

Immune cell subsets in biopsies, quantified by IHC, showed a comparable picture to the cells analyzed by flow cytometry in blister fluid. Administration of prednisolone reduced HLA-DR and infiltrating CD11c (dendritic cells), CD4^+^ (T helper cells), CD1a (Langerhans cells), and CD8^+^ cells (cytotoxic T cells) (**Figure 4**). Time courses of IMQ and prednisolone effects were comparable between biopsy and blister-derived immune cells.

### Oral corticosteroids suppress IMQ-driven cytokine responses in blister exudate and whole blood cultures

**Figure 5:**
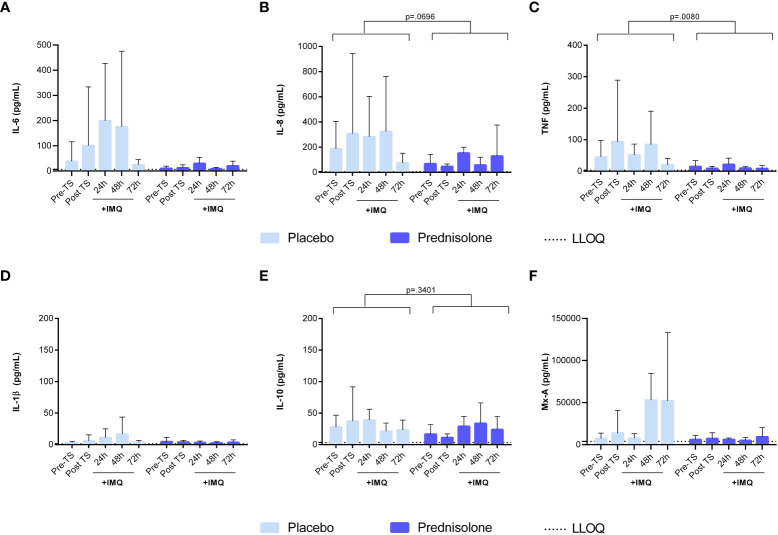
Prednisolone suppresses the NF-κB driven cytokines and IRF7 driven response in blister fluid. **(A)** IL-6 **(B)** IL-8 **(C)** TNF **(D)** IL-1b **(E)** IL-10 **(F)** Mx-A. Cytokine concentrations in blister fluid were analysed by MSD and Mx-A by ELISA. Data are presented as mean ± SD. For IL-6, IL-1b and Mx-A no statistical model was applied as the majority of the values were <LLOQ in the prednisolone group. ELISA, enzyme- linked immunosorbent assay; IL, interleukin; LLOQ, lower limit of quantification; MSD, meso scale discovery; TNF, tumor necrosis factor; TS, tape stripped.

In addition to the dermal cellular response, cytokine levels were analyzed in the blister exudate to evaluate of NF- κB- and IRF7-driven responses. IMQ application induced a mild IL-6 response at 24 and 48 h (**Figures 5A, B**) but had no clear effect on the levels of NF- κB-driven cytokines IL-1β IL-8 and IL-10 (**Figures 5C–E**). IMQ increased the Mx-A concentration in the blister fluid, with a peak at 48 and 72 h (**Figure 5F**), indicating activation of the IRF7 pathway. Prednisolone treatment reduced the levels of NF-κB-driven cytokines (IL-6, IL-8, and TNF), IL-1β, and Mx-A (**Figures 5A–C, F**). No formal statistical analysis for cytokines IL-6, IL-1β, and Mx-A was conducted for the contrast placebo versus prednisolone, because the immune suppression by prednisolone was so strong that most cytokine levels were below the LLOQ. ASC and IFN-γ concentrations in blister fluid are very low, and therefore, have not been reported.

**Figure 6:**
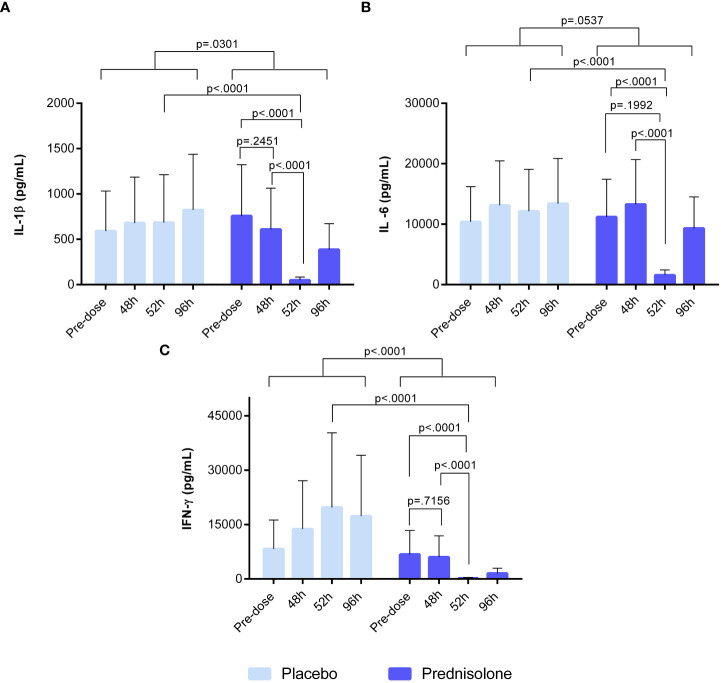
Ex-vivo whole blood stimulated with 20 μg/ml IMQ. **(A)** IL-1b **(B)** IL-6 **(C)** IFN-g. Cytokine concentrations in blood were analysed by V-plex and MSD. Pre-dose time point refers to sample taken prior to prednisolone/placebo dosing. The time point 48h refers to sample taken at 48h after initial dose but before the 5th dose of prednisolone/placebo. Sample taken at 52h refers to time point 4 hours after 5th dose. At 96h after prednisolone/ placebo, the sample was taken before the 8th dose. IFN, interferon; IL, interleukin; MSD, meso scale discovery.

Whole blood samples, drawn from study participants at predefined time points, were stimulated with IMQ for the evaluation of *ex vivo* prednisolone activity. Stimulation with IMQ led to an increase in IL-1β, IFN-γ, and IL-6 levels (**Figures 6A-C**), but not in a detectable Mx-A response (data not shown). An overview of the *ex vivo* results, including those of the unstimulated control conditions, is provided in **Table S2**. Overall, prednisolone compared to placebo had a significant effect on IFN-γ and IL-1β concentrations (estimated difference: −86.8%, 95% CI [−94.1%, −70.3%, p <0.0001), −55.8%, 95% CI [−78.8%, −8.3%], p = 0.0301, respectively), but not IL-6 (estimated difference: −44.1%, 95% CI [−69.1%, 1.0%], p = 0.0537). However, prednisolone treatment resulted in a statistically significant reduction in IMQ-driven cytokines (IL-1β, IL-6, and IFN-γ), with a maximum inhibitory effect at 52 h post-administration, with an estimated difference of −92.9%, 95% CI [−96.8%, −84.3%], p <0.0001, estimated difference: −87.1%, 95% CI [−93.2%, −75.7%], p <0.0001; estimated difference: −99.0%, 95% CI [−99.6%, 97.4%], p <0.0001, respectively. Interestingly, prednisolone had no effect on IMQ-induced response at time points 48 and 96 h. No statistical analysis was performed on the IP-10 concentration, given that more than 60% of the samples were above the ULOQ.

## Discussion

This study aimed to characterize the effects of orally administered prednisolone on TLR7-driven immune responses using *in vivo skin* and *ex vivo* whole-blood IMQ challenges. Prednisolone significantly suppressed the objectified transient clinical response to IMQ (imaging-based perfusion and erythema) as well as clinically graded erythema. An interesting finding was the inhibitory effect of prednisolone on epidermal thickness measured by optical coherence tomography, a noninvasive technique that generates 2D images of tissue microstructure. In a psoriasiform murine model, an abundance of infiltrating cells resulted in a significant increase in epidermal thickness (10). This is in line with our study, which showed an IMQ-driven increase in epidermal thickness at 48 h, suggesting the initiation of an inflammatory response.

To our knowledge, this is the first clinical study to examine the effect of prednisolone on TLR7-driven cellular and cytokine responses *in vivo*. Previous studies have evaluated the local immune response following topical IMQ application. In these studies, dermal cellular and cytokine responses to IMQ were characterized using immunohistochemistry and qPCR. IMQ treatment upregulated chemokines (IP-10), pro-inflammatory cytokines (IL-6), and interferons (Mx-A and IFN-γ). Elevated CD14, CD1a, CD11c, CD4+, and CD8+ cell numbers were observed following IMQ application on tape-stripped skin, peaking at 48- and 72-hour post-dose, comparable to the IHC results in this study (6, 7). However, the current study also evaluated IMQ-driven cellular responses in the skin by flow cytometric analysis of suction blister exudate, providing a more quantitative impression of the inflammatory response. Upon IMQ treatment, a mild influx of NK cells, dendritic cells, and classical monocytes was observed. Oral administration of prednisolone fully suppressed this cell infiltration, confirming the strong anti-inflammatory activity of the compound. Notably, no traces of neutrophils were found in blister fluid, indicating that the applied IMQ regimen did not drive neutrophil attraction, which contradicts earlier preclinical findings, following topical IMQ treatment for 5–6 consecutive days in mice (11). This results in an influx of neutrophils that accumulate beneath the stratum corneum. The absence of neutrophil infiltration in our study may be explained by the duration of IMQ application. The current duration of 48 h IMQ application might not be sufficient to initiate neutrophil influx; therefore, a prolonged application is suggested to align more with the duration in preclinical studies. In contrast to IMQ, LPS drives an acute and strong innate immune response characterized by an influx of neutrophils (peaking at 10 h), monocytes, and NK cells (peaking at 24 h post injection) (8).

TLR7 activation generally drives IRF and NF-κB signaling, playing an important role in the recruitment of immune cells to the dermis (12). Topical application of IMQ resulted in a clear increase in Mx-A concentrations in the blister exudate at 48 and 72 h, indicative of IRF-mediated production of type 1 interferons. Although it is expected to initiate NF- κB signaling and cytokine production (12–14), only a mild IL-6 response was observed, whereas other NF- κB-driven cytokine responses (IL-8, IL-1β, and IL-10) were limited or absent. The relatively mild inflammatory response at the molecular level may be explained by a potentially limited IMQ exposure, related to the partial delivery of applied IMQ to the dermal tissue. Alternatively, the low NF- κB responses may be explained by the timing of the sampling: the innate immune system is activated in phases, with the initial phase resulting in the secretion of proinflammatory cytokines generally occurring within 24 h (5, 15, 16). It is possible that our first post-IMQ blister time point was too late to detect the early innate immune response driven by IMQ. Lastly, it is possible that IMQ in this formulation and in the current regimen, for these types of innate immune challenges, is simply a weak immune agonist compared to LPS. This is also supported by the relatively mild cellular responses observed. Despite the small cytokine responses, a clear effect of prednisolone treatment was observed: Mx-A, IL-6, TNF, and IL-1β responses to IMQ were significantly lower in prednisolone-treated volunteers (although for Mx-A, IL-6, and IL-1β, no formal p-value could be calculated given the substantial number of samples with cytokine levels below the limit of quantification in the prednisolone-treated group). For future studies, the abovementioned readouts can be used to evaluate the effect of IMQ.

Interestingly, oral prednisolone and topical clobetasol treatment did not significantly suppress dermal cytokine responses in an earlier human challenge study that applied intradermal LPS injections, driving TLR4-mediated responses (8). This contrasts with the effects of prednisolone on the IMQ-driven cytokine responses observed in the current study. The difference in treatment response might be explained by the more pronounced dermal cytokine response driven by LPS (~5- to 100-fold) compared to IMQ. This difference in challenge response size, also reflected at the cellular level, may be explained by the different routes of administration—intradermal for LPS versus topical for IMQ—leading to differences in intradermal concentrations. Furthermore, LPS may act as a potent immune agonist. The argument that a stronger immune response is more difficult to counteract by corticosteroids is contradicted by the efficient suppression of LPS-driven skin perfusion, erythema, and local cell attraction by prednisolone and clobetasol. Therefore, the successful suppression of IMQ-driven cytokine responses by prednisolone, versus the poor suppression of LPS-driven responses of the same cytokines, should be investigated at the physiological level: the difference between TLR4 and TLR7 signaling.

Finally, the *ex vivo* pharmacological activity of prednisolone was monitored using whole-blood IMQ. Prednisolone treatment resulted in a significant reduction of IFN-γ, IL-1β, and IL-6 release, but only when measured at 52 h after initiation of prednisolone treatment, which was 4 h after the previous prednisolone intake. Cytokine release was not suppressed 48 and 96 h after the first prednisolone administration. This may be explained by the pharmacokinetic profile of prednisolone which is complex in humans (17). Prednisolone is rapidly absorbed and available between 80% and 100% after oral intake. The plasma concentration peaks 1 to 2 h after administration and the corresponding half-lives vary between 2.5 and 6.6 h and are dose-dependent (18, 19). At 48 and 96 h, shortly before the next prednisolone dose, the suppressive effect of prednisolone was negligible because the systemic concentration of the drug was considered low. Of interest, the reductions in cytokine levels in blister fluid are less dependent on the pharmacokinetic profile of prednisolone, which is concordant with the literature describing that no relationship has been demonstrated between prednisolone concentration in blood and therapeutic effect (20, 21). This discrepancy between the systemic drug activity measured in whole blood cultures *ex vivo* and the peripheral drug effect evaluated in skin *in vivo* underlines the value of *in vivo* human immune challenges such as the topical IMQ challenge for evaluation of drug effects.

In this clinical study, we successfully demonstrated that orally administered prednisolone at a conventional clinical dose suppresses IMQ-induced skin inflammation in healthy volunteers, which underlines the potential value of this cutaneous challenge model for future clinical pharmacology studies with novel anti-inflammatory compounds targeting the TLR7 pathway. In these studies, prednisolone can be used as a benchmark.

## Data availability statement

The original contributions presented in the study are included in the article/**Supplementary Material**. Further inquiries can be directed to the corresponding author.

## Ethics statement

The studies involving human participants were reviewed and approved by Stichting Beoordeling Ethiek Biomedisch Onderzoek. The patients/participants provided their written informed consent to participate in this study.

## Author contributions

MM devised the project and main conceptual ideas together with SA, TB, NT, and MJ. SA, TB, MJ, and MM worked out technical details and study design. SA and TB coordinated the clinical trial. PH coordinated and performed bioanalysis. NK carried the medical responsibility. SA, TB, MM, MJ, CH, and PH analyzed and interpreted data. MK carried out statistical analysis. SA, TB, MJ, and MM wrote the manuscript. All authors listed have made a substantial, direct, and intellectual contribution to the work and approved it for publication.

## Supplementary material

Supplementary Figure 1 Clinical erythema assessment by physician.

Supplementary Figure 2 Gating strategy used for flow cytometry analysis of the blister exudate.

Supplementary Table 1 Overview of number of samples per assessment.

Supplementary Table 2 Overview of whole blood *ex vivo* results stimulated and unstimulated with IMQ 20µg/ml.
