# Supplementary material for: Oral prednisolone suppresses skin inflammation in a healthy volunteer imiquimod challenge model
Source: Front Immunol. 2023 Jul 20;14:1197650. doi: 10.3389/fimmu.2023.1197650 (PMC10400434; doi:10.3389/fimmu.2023.1197650)
Supplement: Supplementary Figure 1 — Clinical erythema assessment by physician. [file Presentation_1.pptx]

## Slide 1
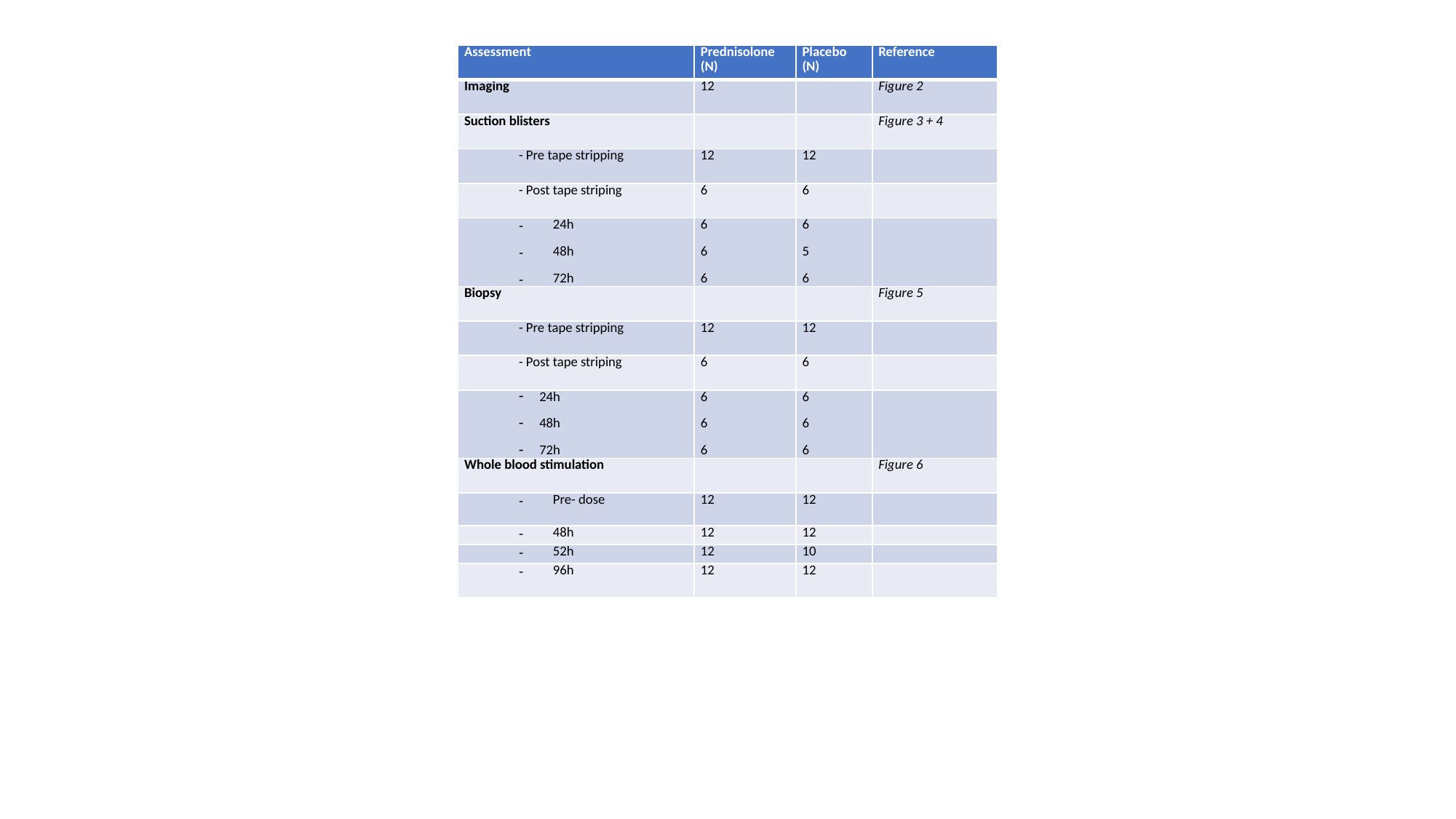

| Assessment | Prednisolone (N) | Placebo (N) | Reference |
| --- | --- | --- | --- |
| Imaging | 12 | | Figure 2 |
| Suction blisters | | | Figure 3 + 4 |
| - Pre tape stripping | 12 | 12 | |
| - Post tape striping | 6 | 6 | |
| 24h 48h 72h | 6 6 6 | 6 5 6 | |
| Biopsy | | | Figure 5 |
| - Pre tape stripping | 12 | 12 | |
| - Post tape striping | 6 | 6 | |
| 24h 48h 72h | 6 6 6 | 6 6 6 | |
| Whole blood stimulation | | | Figure 6 |
| Pre- dose | 12 | 12 | |
| 48h | 12 | 12 | |
| 52h | 12 | 10 | |
| 96h | 12 | 12 | |

## Slide 2
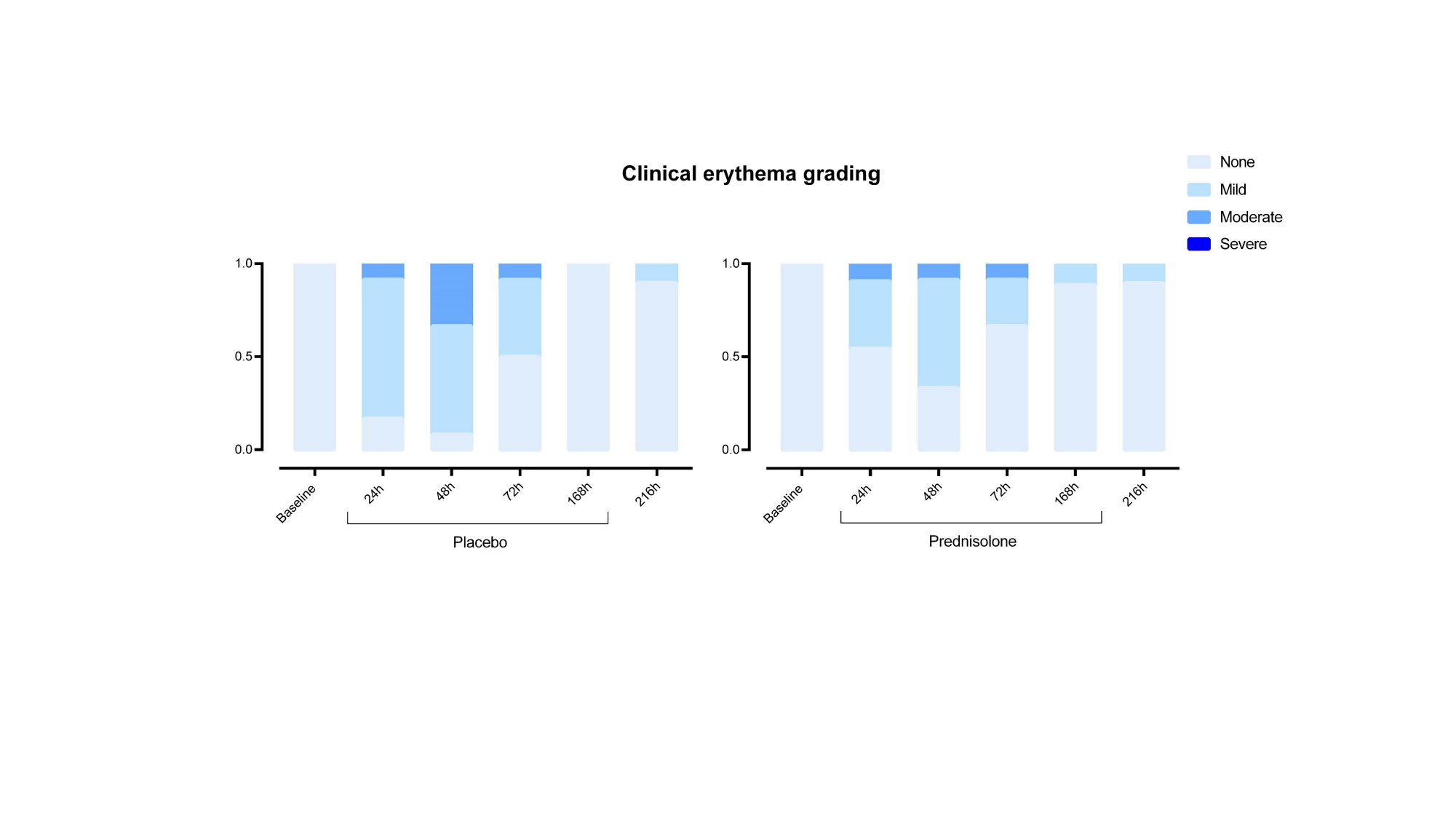

## Slide 3
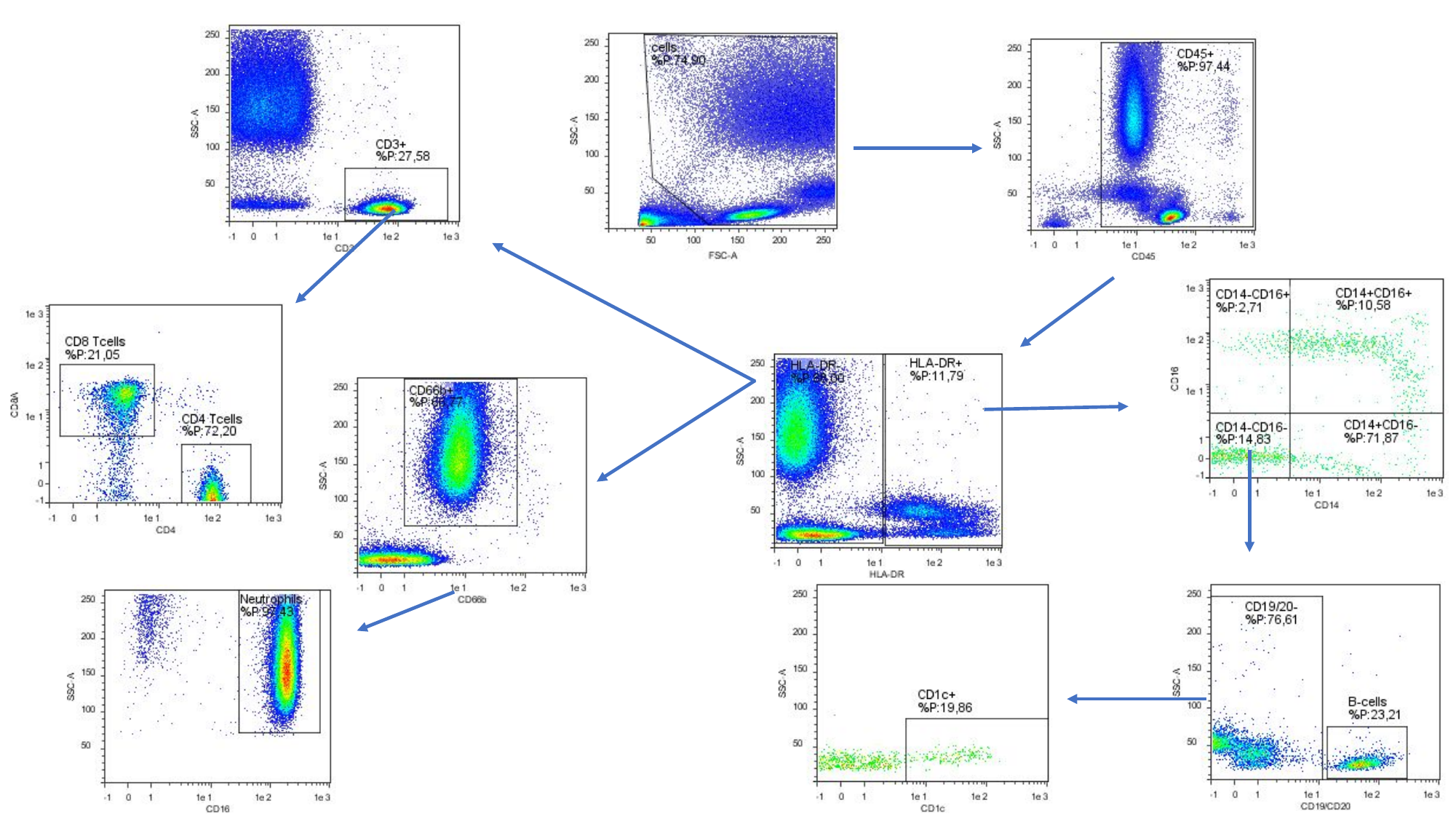

## Slide 4
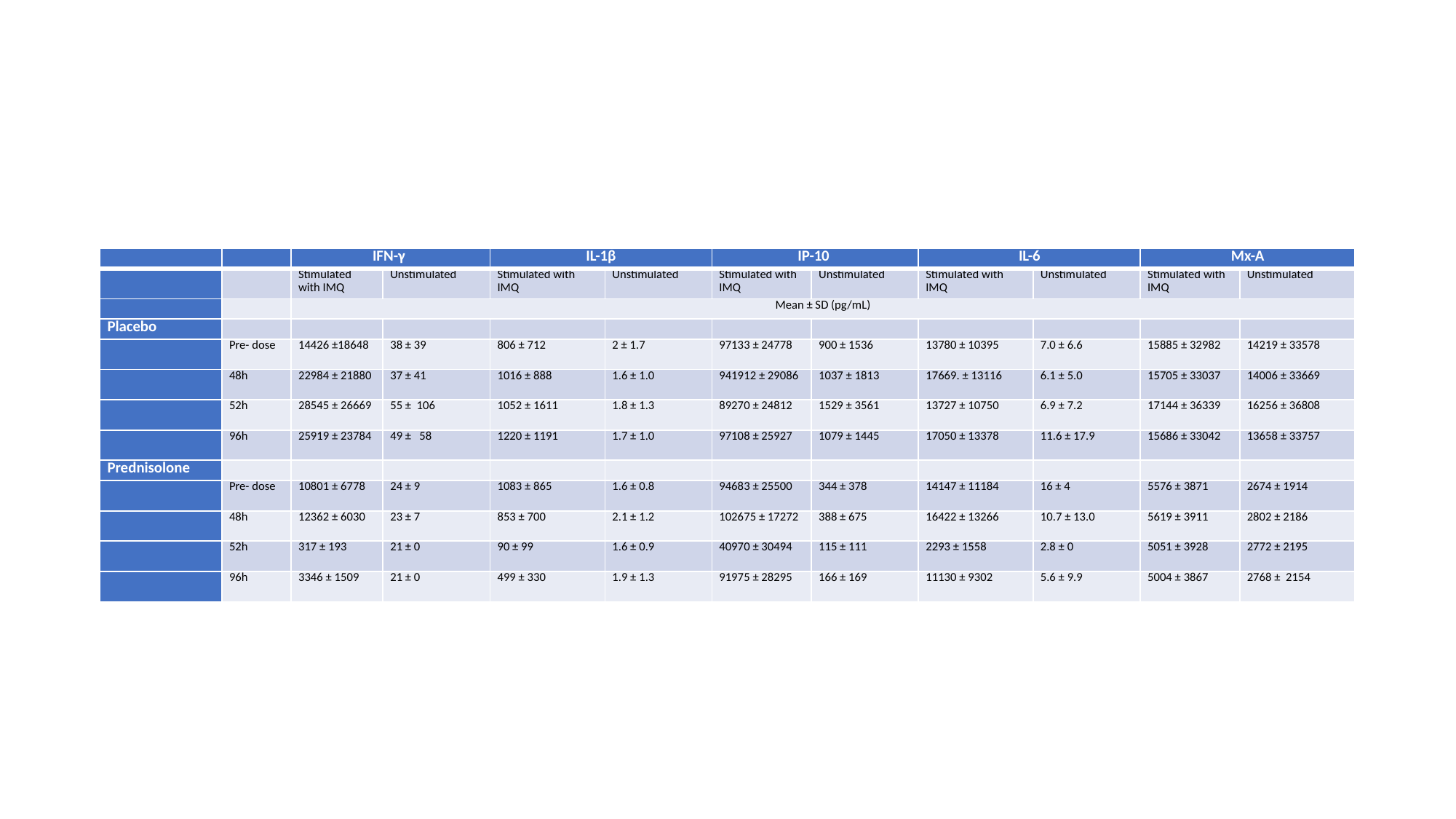

| | | IFN-γ | | IL-1β | | IP-10 | | IL-6 | | Mx-A | |
| --- | --- | --- | --- | --- | --- | --- | --- | --- | --- | --- | --- |
| | | Stimulated with IMQ | Unstimulated | Stimulated with IMQ | Unstimulated | Stimulated with IMQ | Unstimulated | Stimulated with IMQ | Unstimulated | Stimulated with IMQ | Unstimulated |
| | | Mean ± SD (pg/mL) | | | | | | | | | |
| Placebo | | | | | | | | | | | |
| | Pre- dose | 14426 ±18648 | 38 ± 39 | 806 ± 712 | 2 ± 1.7 | 97133 ± 24778 | 900 ± 1536 | 13780 ± 10395 | 7.0 ± 6.6 | 15885 ± 32982 | 14219 ± 33578 |
| | 48h | 22984 ± 21880 | 37 ± 41 | 1016 ± 888 | 1.6 ± 1.0 | 941912 ± 29086 | 1037 ± 1813 | 17669. ± 13116 | 6.1 ± 5.0 | 15705 ± 33037 | 14006 ± 33669 |
| | 52h | 28545 ± 26669 | 55 ± 106 | 1052 ± 1611 | 1.8 ± 1.3 | 89270 ± 24812 | 1529 ± 3561 | 13727 ± 10750 | 6.9 ± 7.2 | 17144 ± 36339 | 16256 ± 36808 |
| | 96h | 25919 ± 23784 | 49 ± 58 | 1220 ± 1191 | 1.7 ± 1.0 | 97108 ± 25927 | 1079 ± 1445 | 17050 ± 13378 | 11.6 ± 17.9 | 15686 ± 33042 | 13658 ± 33757 |
| Prednisolone | | | | | | | | | | | |
| | Pre- dose | 10801 ± 6778 | 24 ± 9 | 1083 ± 865 | 1.6 ± 0.8 | 94683 ± 25500 | 344 ± 378 | 14147 ± 11184 | 16 ± 4 | 5576 ± 3871 | 2674 ± 1914 |
| | 48h | 12362 ± 6030 | 23 ± 7 | 853 ± 700 | 2.1 ± 1.2 | 102675 ± 17272 | 388 ± 675 | 16422 ± 13266 | 10.7 ± 13.0 | 5619 ± 3911 | 2802 ± 2186 |
| | 52h | 317 ± 193 | 21 ± 0 | 90 ± 99 | 1.6 ± 0.9 | 40970 ± 30494 | 115 ± 111 | 2293 ± 1558 | 2.8 ± 0 | 5051 ± 3928 | 2772 ± 2195 |
| | 96h | 3346 ± 1509 | 21 ± 0 | 499 ± 330 | 1.9 ± 1.3 | 91975 ± 28295 | 166 ± 169 | 11130 ± 9302 | 5.6 ± 9.9 | 5004 ± 3867 | 2768 ± 2154 |
